# The Occurrence of Mandible Brown Tumor Mimicking Central Giant Cell Granuloma in a Case Suspicious of Primary Hyperparathyroidism—Troublesome Diagnostic Dilemmas

**DOI:** 10.3390/diagnostics15162038

**Published:** 2025-08-14

**Authors:** Kamil Nelke, Klaudiusz Łuczak, Maciej Janeczek, Marcelina Plichta, Agata Małyszek, Małgorzata Tarnowska, Piotr Kuropka, Maciej Dobrzyński

**Affiliations:** 1 Maxillo-Facial Surgery Ward, EMC Hospital, Pilczycka 144, 54-144 Wrocław, Poland; 2 Department of Biostructure and Animal Physiology, Wrocław University of Environmental and Life Sciences, Kożuchowska 1, 51-631 Wrocław, Poland; 3 Faculty of Chemistry, University of Wroclaw, Fryderyka Joliot-Curie 14, 50-300 Wrocław, Poland; 4 Division of Histology and Embryology, Department of Biostructure and Animal Physiology, Wrocław University of Environmental and Life Sciences, Cypriana K. Norwida 25, 50-375 Wrocław, Poland; 5 Department of Pediatric Dentistry and Preclinical Dentistry, Wrocław Medical University, Krakowska 26, 50-425 Wrocław, Poland

**Keywords:** bone, hyperparathyroidism, mandibular lesion, central giant cell lesion, CBCT

## Abstract

The jaw bones can manifest various cysts and tumors of different origins and etiologies. Any bone lesions lacking any potential odontogenic origin might require more accurate diagnostics, adequate investigation, and careful patient anamnesis. In cases of sharply demarcated radiolucency or mixed radiolucent–radiopaque radiological appearance lesions, they can sometimes extend between the displaced tooth roots or cause their resorption. The scope of cortical bone in radiographic studies might have a different status, and lesions can spread outside of the bone. If no odontogenic feature is present, an additional blood examination for bone markers and calcium–phosphate markers is useful to establish any endocrine-related pathologies. In the primary hyperparathyroidism (PHP), bone blood markers and bone scintigraphy are very useful to establish the possible occurrence of brown tumor. On the other hand, in central giant cell granuloma (CGCG), only a direct tumor lesion biopsy might confirm the diagnosis, where in microscopic evaluation, mostly fibroblasts and secondary cells have multinucleated giant cells along with some accessory cells like macrophages, dendrocytes, and other endothelial cells. Because both lesions can have similar clinical and radiological appearances and unclear borders, with different shapes, sizes, and symptoms, it is quite important to compare both clinical and radiological patient characteristics. The authors aim to present how radiological studies alone can easily lead to lesion misdiagnosis. They also aim to emphasize how local treatment methods without advanced microsurgical reconstruction can, in some cases, improve patient outcomes.

**Figure 1:**
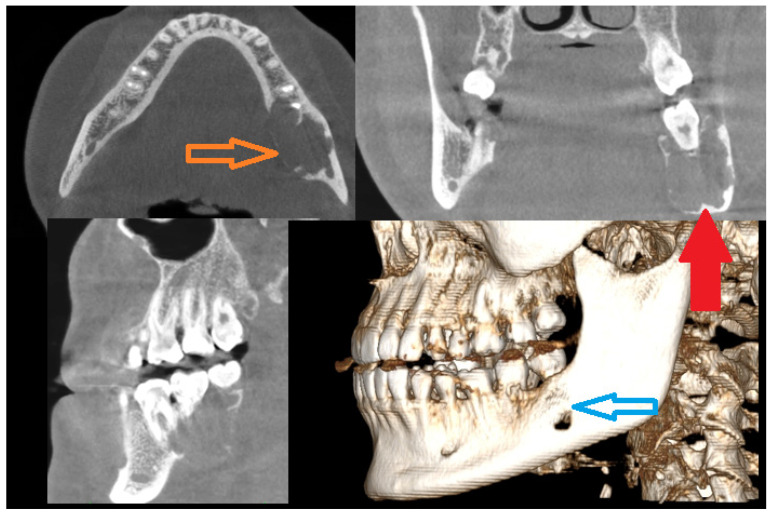
Cortical bone erosion and expansion might be a sign of a locally aggressive lesion (orange arrow, blue arrow). On the other hand, additional symptoms like tooth resorption, no signs of pathological fracture, and no lower lip numbness (lacking Vincent’s sign) could be helpful in tumor diagnostics. Irregular solid like mass with cortical spread and teeth resorption should be scheduled for a biopsy at first, since radiologically many primary, secondary, and even metastatic lesions can have a similar appearance [1,2,3]. Good CBCT (cone-beam computed tomography) improves the diagnostics when dental root status and possible odontogenic-related lesions are either confirmed or excluded. Symptoms alone do not influence the diagnostics; however, some atypical general coexisting patient conditions related to neurological symptoms (depression, tiredness, loss of concentration), excessive urination, loss of appetite and abdominal pain (nausea, vomiting), osteoporosis, muscle pain, and weakness followed by kidney stones and joint pains might be general symptoms manifesting primary hyperparathyroidism (PHP) cases, where some jaw tumors (brown tumor (BT), also known as osteitis fibrosa cystica) also occur [1]. In atypical jaw bone tumors, BT manifests in radiologically well-defined radiolucent areas with non-corticated borders and heterogenous radiolucent or radiopaque–radiolucent areas in the mandible bone. Secondly, teeth displacement, gum swelling, atypical periodontal status, and pain are present. PHP is related to endocrine disturbances caused by parathyroid hormone (PTH) overproduction. In most cases, the occurrence of a single adenoma, multiple adenomas, or, rarely, a carcinoma (1%) in the parathyroid gland is the main cause, with some female predominance. SPECT-CT (single photon emission computed tomography) and estimation of blood markers such as serum calcium, PTH levels, magnesium, triiodothyronine (T3), thyroxine (T4), thyroid-stimulating hormone (TSH), vitamin D, serum calcium, alkaline phosphatase (ALP), and phosphorus are sufficient to confirm PHP [2]. Magnesium, iron, or other ion levels are rarely investigated, but their role has never been fully elucidated. Quite often, parathyroidectomy causes all BTs to decrease, and the residual tumor sometimes requires some kind of surgical approach. On the other hand, a giant cell lesion of the jaw bones can also be present without any coexisting endocrine pathologies with a similar radiological and clinical appearance. A CGCG, central giant cell granuloma, is a benign, nonodontogenic lesion of the jaw, osteoclastic in origin, with unknown etiology mostly with asymptomatic painless swelling of the jaw bones (similar to that indicated by the red arrow). More advanced lesions tend to displace teeth and cause their resorption, and there is also a tendency for invading the oral mucosa after cortical bone thinning (red arrow) and perforation (blue arrow), with some bluish/brownish discoloration that might appear. Radiologically, they mostly present as radiolucent areas between the roots with a slow expansile character without any clear calcifications within and not uncommon extra-cortical bone spread (the approx. size of the longest axis of the CBCT image lesion was 45 × 34 × 28 mm) [1,2,3]. CGCG is mostly diagnosed through histopathological sample examination. In the presented case, this lesion was growing slowly over a long period of time. The radiological investigation was inconclusive, the blood examination for calcium–phosphate markers was not significant in this case, and biopsy was not indicative of the diagnosis of any odontogenic tumors, cancers, or endocrine bone-related pathologies; however, a non-specific cystic appearance was found, and because of the following, a decision was made to perform radical surgery.

**Figure 2:**
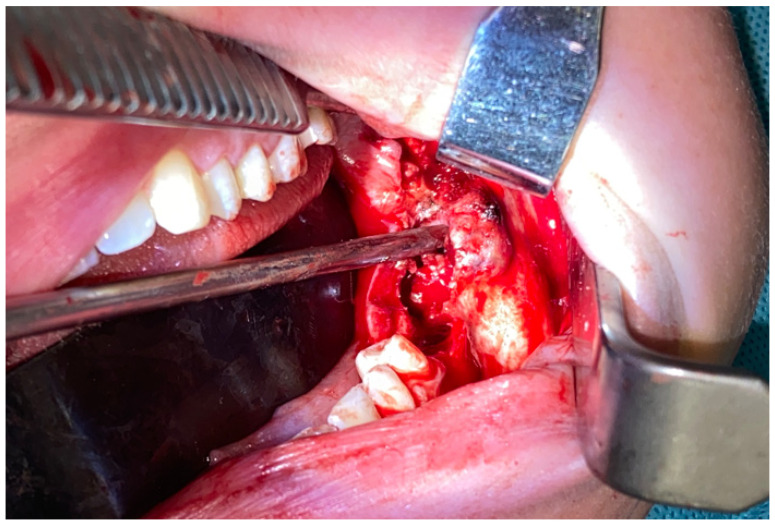
Initial diagnosis excluded any odontogenic cysts and tumors. Endocrine disorders were excluded; however, the value of PTH was in its upper range limits, which was atypical and not conclusive of a BT in PHP. The biopsy and evaluation of calcium–phosphate markers were not significant either. Because of lesion growth, the possibility of pathological fracture, secondary bone disfigurement, and perhaps a necessity in the future for a free-flap, the decision was made to undergo surgery while some of the patient’s own bone was still present. A possible option for a conservative approach was avoided to decrease the risk of any possible pathological mandibular fracture during this bone tumor progression. Because of the possibility of reducing the scope of surgery and maintaining a healthy lower mandibular bone border, it was possible to use an alternative approach different from the typical en-block resection with microsurgical reconstruction. Secondly, because of the patient’s young age and her expectancy for a good and less invasive outcome and avoidance of a facial scar, an intraoral approach was used. Because of the location, a superior-based mandibular body marginectomy with bone ostectomy, teeth removal (36–38), and resection of purple-like gum tumor with prophylactic stable bone osteosynthesis of the mandible was scheduled (two Medartis 2.0 titanium plates with seventeen screws 2.0 7/8 mm, Medartis, Basel, Switzerland). Intraorally, the entire polycystic lesion had a solid appearance without any mineralization or cystic appearance, but in both the radiological and clinical examination, the lesion had an expansile behavior. The lower inferior alveolar nerve was displaced within the tumor’s inferior aspect and was not untacked during surgery. The entire bone defect was filled with allogenic bone grafts (20 cm3 of bone) from the local bio-bank and additional bone marrow aspirate concentrate (BMAC) from the iliac crest, with blood mixed together. Three collagen membranes (Matrix Flex, Athena Marketing, Vadodara, Gujarat, India) were placed on the lingual aspect of the bone and sutured with 4.0 Prolene sutures (Prolene—B.Braun, Melsungen, Germany) to the flaps to ensure more stability to the allogenic bone. Four additional IMF screws (intramaxillary fixation, Medartis, Basel, Switzerland) were applied to avoid any secondary trauma.

**Figure 3:**
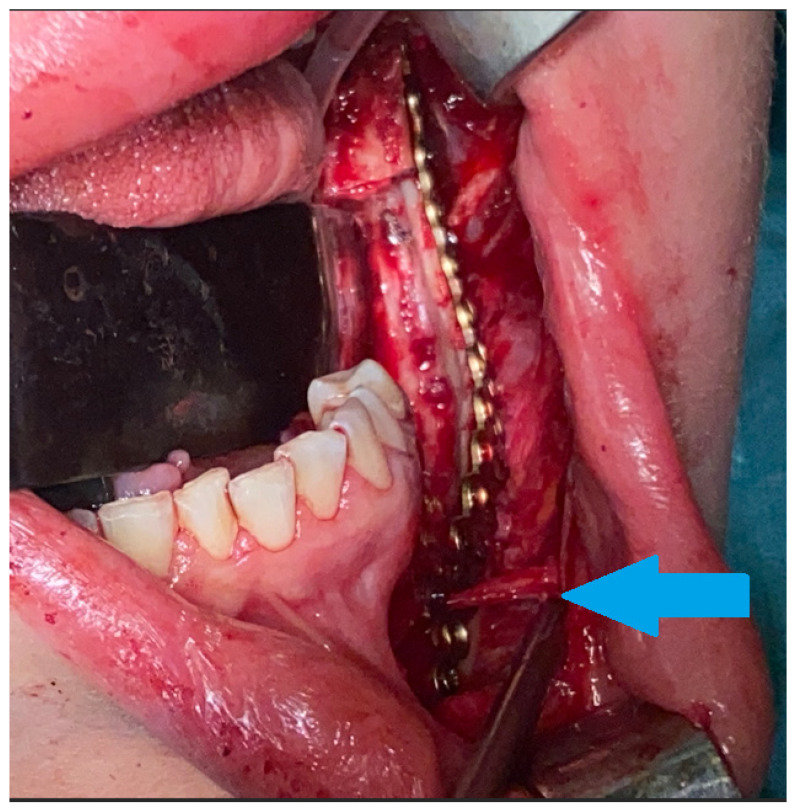
Intraoperative view after reconstruction and superior mandibular body marginectomy. A thin layer of intact lower mandibular border was kept and used for stable bone osteosynthesis (titanium 2.0 plates, Medartis, Basel, Switzerland) to prevent any secondary pathological fracture. The mental nerve was spared and safely retracted away (blue arrow). Surgical ostectomy was made with burrs and Lindemann drills. We avoided any unnecessary segmental resection, skin scarring, and the necessity for a bigger procedure, meaning that this surgical procedure was performed with a good overall success rate. CGCG cannot be diagnosed in any laboratory testing, and histopathology sometimes might be insufficient, where it can be misdiagnosed as BT, while radiological appearance might mimic other odontogenic cysts or tumors. On the other hand, fully grown and active BT is easily diagnosed with blood examination for calcium–phosphate markers alone. Perhaps very early in the BT formation stage, its prolonged slow growth or atypical manifestation and close relation between BT and CGCG can lead to such diagnostic dilemmas. It is worth noticing that small local lesions can be treated with nonsurgical means or local minimally invasive surgical approaches [1,2]. Some studies advice local tumor injections of calcitonin, interferon alpha, or intratumor steroids; however, the success rates vary in the known literature [3,4,5]. On the other hand, the scope of more aggressive and advanced lesions might require different surgical approaches, and in the literature, its possible outcomes vary from bone curettage and ostectomy to radical segmental osteotomies with immediate bone reconstructions or surgical debulking. Based on the following, the scope of surgery is case-driven and depends greatly on the tumor size, shape, location, and the adjacent bone condition. The presented case outlines how such a big lesion was treated by the authors with a great overall surgical success. Because of some radiological similarities between BT and CGCG, careful patient examination is mandatory, and additional bone markers should always be evaluated, and even repeated over time. It is quite important to identify any PHP or a simple CGCG lesion in good early timing to improve the patient’s general condition. Without an improved excisional biopsy in order to examine the entire specimen, its highly possible that the patient could suffer for a long time because of general status worsening caused by the effects of PHP calcium–phosphate on multiple organ systems over various intensities and time frames, causing, for example, bone osteoporosis, kidney stones, nephrocalcinosis, hypertension, cardiovascular disturbances, neuropsychiatric symptoms, pancreatitis, and muscle and joint weakness, among others [3,4,5].

**Figure 4:**
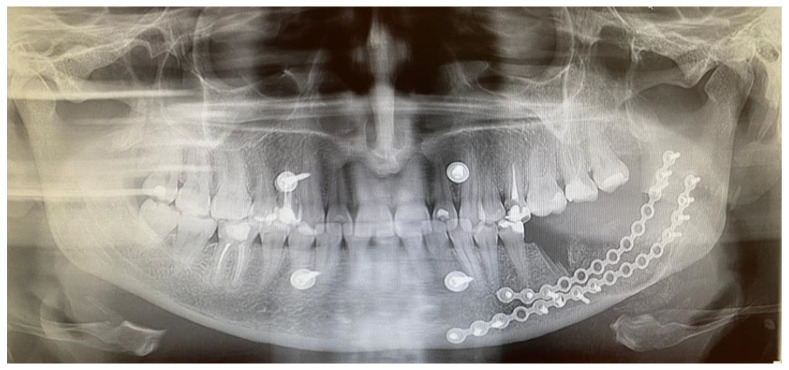
An immediate early panoramic radiograph with very good and stable outcomes. After tumor removal, ostectomy, local resection, and reconstruction with allogenic bone graft and titanium plates, the result is very good. Because a very thin healthy bone layer was remaining, additional IMF—intramaxillary—screws (titanium 2.0 plates, Medartis, Basel, Switzerland) were used to maintain stable occlusion, avoid any fracture, and decrease the possibility of any wound dehiscence and irritation. It is worth noting and remembering that CGCG should be differentiated from the following lesions and tumors: brown tumors in PHP, peripheral form of CGCG (PGCG), giant cell tumor, lesions from cherubism, non-ossifying fibromas, and ABCs—aneurysmal bone cysts—or even ameloblastoma [4,5]. Radiological differentiation should also include myxoma/odontogenic-myxoma (OM), ameloblastic fibroma (AF), and ossifying fibroma (OsF); however, the additional presence of calcifying and bone masses inside the lesions, with an irregular border and lesion shape, improves the diagnosis. Some atypical cyst formation, spread, and occurrence in the jaw bone might also mimic other tumors or lesions, which is why it is crucial to evaluate each atypical case carefully [6]. This case underlines how similar radiological appearances can be seen in various different tumors, and how not only a biopsy but bone makers with SPECT examination can improve the diagnostics. Each BT and CGCG can be misdiagnosed, which is why improved histopathology is important w a larger sample. This case excluded PHP; however, more extensive histopathological evaluation concluded an occurrence of typical brown tumor characteristics for a long but slow growth, but without any significant correlation between the endocrine and calcium–phosphate markers, which is quite unusual.

**Figure 5:**
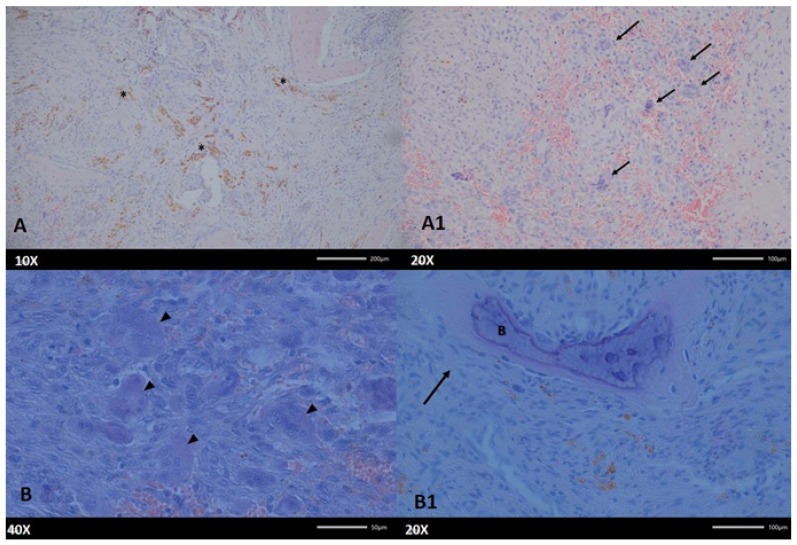
In hyperparathyroidism, the bone undergoes dramatic and characteristic histological changes that are most conspicuous in advanced disease, particularly in the form of osteitis fibrosa cystica. CGCG lesions could mimic PHP-related lesions, where the level of PTH influences the scope of each bone lesion. CGCG could often grow slowly and progress over time with asymptomatic features. The changes presented in the histopathological evaluation correspond with the radiological changes; however, the elevated PTH levels could be easily misinterpreted. The material for the study was collected by the study’s authors. (**A**) The newly formed connective tissue contains numerous aggregations of hemosiderin (asterisks, for example), which provides the tissue with a specific brown color. (**A1**) In the connective tissue, numerous multinucleated cells and extravasated erythrocytes are noted. Hematoxylin and eosin staining (H&E). Magnification A—100×; B—200×. On (**B**) multinucleated cells (arrowheads) surrounded by fibrous connective tissue containing remnants of poorly mineralized bone, trabeculae (B) surrounded by fibroblasts synthesizing collagen fibers (arrow) are visible on the picture (**B1**). Hematoxylin and eosin staining (H&E). Magnification A—400×; B—200×.

**Figure 6:**
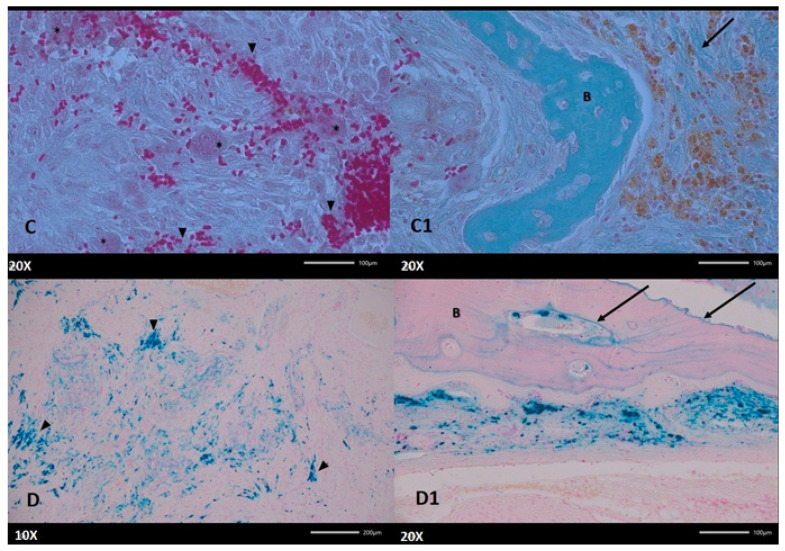
(**C**) Bone tissue is gradually converted into fibrous connective tissue in which numerous extravasated erythrocytes (arrowheads) and multinucleated cells (asterisk) are visible. (**C1**) Remnants of poorly mineralized bone trabeculae (B) are surrounded by deposits of hemosiderin present in macrophages surrounded by collagen fibers (arrow). Masson Goldner staining. Magnification 200×. (**D**) The newly formed connective tissue contains numerous deposits of iron (Fe^3+^) derived from hemosiderin, visualized as blue clusters (arrowheads, for example). (**D1**) Iron (Fe^3+^) deposits are mainly localized in macrophages (blue color), but positive reactions are also detected in bones (B) in the form of irregular blue lines (arrows), which is caused by their direct, long-term deposition on bones. The asterisk points to a blood vessel with visible blue-stained pericytes. Mallory’s method. Magnification (A) 100×; (B) 200×. In some conditions, without a histopathological browning in the sample and its typical histopathological appearance, a CGCG might be suspected, especially if all endocrine conditions were excluded. Both radiological assessment and blood panel marker evaluations, and even a typical biopsy, might not be sufficient. In this case, a detailed improved histopathological examination with adequate staining was crucial to confirm the final diagnosis and identify this atypical manifestation of a BT and distinguish it from CGCG. Authors like Hussain et al. and Lis et al. confirm that both radiological and clinical manifestation for mandible tumors might be misdiagnosed with many other odontogenic, non-odontogenic, and pseudo-tumor-like pathologies [3,6].

Ongoing bone resorption and microfractures can result in cyst-like spaces that can coalesce into larger cavities. Locally, islands of newly synthesized, poorly mineralized bone, covered by multiple active osteoblasts, can be spotted. In conclusion, these important histopathological findings point out that the presence of these giant cells in a fibrous, hemorrhagic stroma is a key diagnostic feature and may sometimes mimic other giant cell lesions. In the histologic pattern of increased osteoclast activity, fibrous tissue proliferation is noted. Although primarily seen in the context of primary hyperparathyroidism, similar changes may be encountered in cases of secondary or tertiary hyperparathyroidism, albeit with differing clinical contexts. The microscopic findings—resorbed trabeculae, peritrabecular fibrous tissue, abundant osteoclast-like cells, and zones of hemorrhage with hemosiderin—are diagnostic clues that help differentiate hyperparathyroid bone disease from other osteolytic conditions. If these findings are correlated with the clinical picture (including hypercalcemia and elevated PTH levels), these histological features become essential to confirming the diagnosis of hyperparathyroidism-related bone disease. Understanding these histological changes is crucial not only for making the diagnosis but also for appreciating why patients with untreated hyperparathyroidism may suffer from bone pain, skeletal deformities, and an increased risk of fractures. The dynamic interplay between resorption and attempted repair in the bone creates a structurally weakened framework that is prone to these clinical complications. On a therapeutic level, addressing the excess PTH—whether by surgical or medical means—can help reverse some of these destructive processes and stabilize the bone architecture [1,2,3,4,5,6].

**Figure 7:**
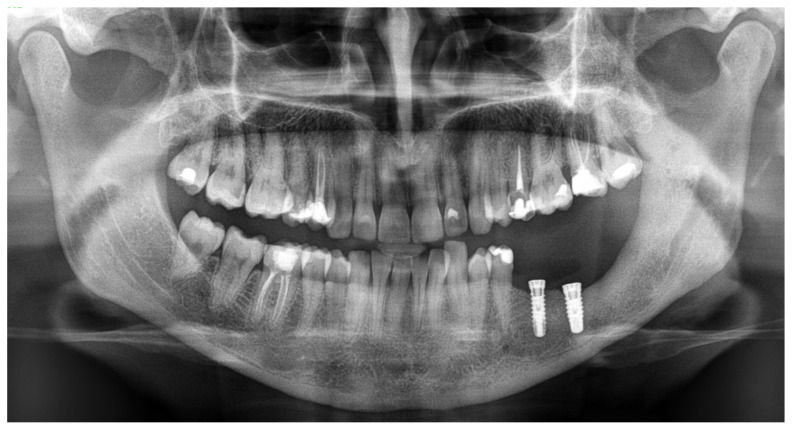
The patient wanted a good and stable outcome because of missing teeth, and a decision was made for dental implant placement in the regenerated left mandibular basis after allogenic bone graft remodeling. During preparation for surgery, titanium plates were removed, and the bone base for dental implants was prepared. Regarding the long-term outcome after a 24-month time frame, the patient is free of disease. This case shows how superior marginectomy with ostectomy and allogenic bone grafts can be a suitable approach when the inferior alveolar bone margin is not involved in the disease. Secondly, no radical segmental bone resection was needed, and no scars are present.

## Data Availability

The datasets used and/or analyzed during the current study are available from the corresponding author upon reasonable request.
